# Friend or Foe? The Varied Faces of Homeostatic Synaptic Plasticity in Neurodegenerative Disease

**DOI:** 10.3389/fncel.2021.782768

**Published:** 2021-12-10

**Authors:** Henry B. C. Taylor, Alexander F. Jeans

**Affiliations:** Department of Pharmacology, University of Oxford, Oxford, United Kingdom

**Keywords:** neurodegeneration, synaptic plasticity, synaptic scaling, Alzheimer’s, Parkinson’s, Huntington’s, amyotrophic lateral sclerosis

## Abstract

Homeostatic synaptic plasticity (HSP) regulates synaptic strength both pre- and postsynaptically to ensure stability and efficient information transfer in neural networks. A number of neurological diseases have been associated with deficits in HSP, particularly diseases characterised by episodic network instability such as migraine and epilepsy. Recently, it has become apparent that HSP also plays a role in many neurodegenerative diseases. In this mini review, we present an overview of the evidence linking HSP to each of the major neurodegenerative diseases, finding that HSP changes in each disease appear to belong to one of three broad functional categories: (1) deficits in HSP at degenerating synapses that contribute to pathogenesis or progression; (2) HSP induced in a heterosynaptic or cell non-autonomous manner to support the function of networks of which the degenerating synapses or cells are part; and (3) induction of HSP within the degenerating population of synapses to preserve function and to resist the impact of synapse loss. Understanding the varied manifestations of HSP in neurodegeneration will not only aid understanding mechanisms of disease but could also inspire much-needed novel approaches to therapy.

## Introduction

The ability of physiological systems to respond to and resist perturbations is vital to their continued normal and healthy functioning. In addition to the well-understood homeostatic systems that allow for the maintenance of key variables such as temperature or blood pressure within set bounds, it has become apparent that there are a variety of mechanisms responsible for maintaining the function of neuronal circuits and networks within an appropriate range. The best-studied of these mechanisms are those of synaptic scaling, otherwise known as homeostatic synaptic plasticity (HSP), which adjust synaptic strengths across either the whole neuron or a functionally relevant subregion (e.g., dendritic branch) in order to prevent excessive strengthening or weakening of synapses by the potential positive feedback loop created by Hebbian plasticity (Davis, 2013; Vitureira and Goda, 2013). HSP is therefore a critical check that is required to maintain both neuronal health and the fidelity of information transfer (Turrigiano, 2012). HSP utilizes a number of mechanisms that partly overlap with those mediating other forms of synaptic plasticity; while most of these are intrinsic to neurons, under some conditions glial-secreted factors such as TNFα or IL-33 may also be required (Wang et al., 2021). In addition to HSP, a number of other homeostatic mechanisms can also be deployed to stabilise neuronal activity across individual cells and networks. These include regulation of synapse number (Kirov et al., 1999; Wierenga et al., 2006), regulation of intrinsic neuronal excitability (Marder and Goaillard, 2006; Turrigiano, 2011), metaplastic changes to the thresholds for induction of Hebbian plasticity in response to previous synaptic activity (Abraham, 2008) and shifting the balance of excitatory and inhibitory activity (E/I ratio) within networks (Maffei et al., 2004; Gonzalez-Islas and Wenner, 2006).

A number of neurological disease states have been associated with disruption of the mechanisms of HSP, including migraine (Welch, 2003), autism (Nelson and Valakh, 2015), epilepsy (Turrigiano, 2011) and Rett syndrome (Della Sala and Pizzorusso, 2014). HSP dysfunction has also been proposed as a significant factor in the development of schizophrenia (Dickman and Davis, 2009), and it appears to be particularly important in mood disorders occurring either alone or associated with other neurological conditions. Indeed, it is becoming clear that not only does modulation of HSP offer a potentially fruitful therapeutic approach to mood disorder treatment, but some of the most effective current treatments for these disorders actually work *via* this mechanism (Kavalali and Monteggia, 2020). Many of these conditions have in common a tendency towards episodic dysregulation of activity across neuronal networks, which might be expected if key homeostatic regulators of activity are compromised. For example, inherited mutations in the pore-forming subunit of the Ca^2+^ channel Ca_V_2.1, a key presynaptic effector of homeostatic synaptic plasticity (Jeans et al., 2017), can be an inherited cause of either migraine or epilepsy, which result from the aberrant, episodic depression or enhancement of activity, respectively, across networks (Haan et al., 2008).

It is increasingly becoming evident that neurodegenerative diseases constitute part of this group of conditions associated with deficits in HSP. Indeed, it has been recognised for some years that neuronal network-level function is pathologically unstable in several neurodegenerative diseases, potentially accounting for the day-to-day shifts in performance status that characterise many of them (Palop et al., 2006). This mini review will focus on the emerging literature supporting a role for HSP in the major neurodegenerative diseases and in particular highlight an intriguing dichotomy that is becoming apparent. In certain settings, as with non-degenerative neurological diseases, the failure of mechanisms of HSP likely contributes to pathogenesis and progression. Yet in others, HSP has a very different significance as it can be recruited to resist disease progression and confer a degree of protection against the loss of neuronal and network function.

## Alzheimer’s Disease

Alzheimer’s disease (AD) is a substantial and increasing societal burden that is predicted to affect over 100 million people by 2050 (Prince et al., 2013). The disease is thought to be triggered by the pathological accumulation of soluble oligomers of amyloid β (Aβ; Mucke and Selkoe, 2012), which exert a variety of effects on different cell types (De Strooper and Karran, 2016). Alterations in synaptic transmission are among the earliest observed effects, and these include enhanced excitatory activity in cortico-hippocampal networks (Busche and Konnerth, 2016) and impaired Hebbian synaptic plasticity at glutamatergic synapses, specifically a marked attenuation of long-term potentiation (LTP) and facilitation of long term depression (LTD; Walsh et al., 2002; Hsieh et al., 2006; Shankar et al., 2008; Li et al., 2009, 2011). Both of these phenomena precede the appearance of pathology and are considered to be the key cellular substrates of early cognitive impairment in AD.

There is a substantial body of evidence in support of the idea that deficiencies in HSP might contribute to the initiation and/or progression of AD pathogenesis. At the most fundamental level, functional dysregulation of a variety of key protein effectors of various aspects of HSP has been associated with the development of AD. These proteins include β-secretase and presenilin 1, which are responsible for different steps in the cleavage pathway that regulates the production of Aβ from the amyloid precursor protein (APP). Changes in the activity of either due to AD-causative mutations in the protein itself (presenilin 1), or mutations in the sequence within APP that is recognised and cleaved by the protein (β-secretase), are associated with enhanced production of Aβ and consequently AD pathogenesis (Hutton and Hardy, 1997; Zhang et al., 2017). Presenilin 1 has also been shown to be required for Akt-dependent homeostatic upscaling of synaptic strength following chronic blockade of action potential firing with TTX (Pratt et al., 2011), while β-secretase is essential for homeostatic increases in synaptic strength in the visual cortex following dark exposure, as well as for normalization of synaptic transmission by subsequent exposure to light (Petrus and Lee, 2014). These observations might be at least partly explained by the role that Aβ itself appears to play in HSP since a recent study has demonstrated that soluble, secreted Aβ, but not the parent protein APP nor other APP cleavage products, mediates the homeostatic upscaling of synaptic strength following TTX treatment in cultured neurons (Galanis et al., 2021). While this study suggests that Aβ may act in this context *via* postsynaptic mechanisms, other work indicates that it can regulate presynaptic function in response to chronically altered synaptic activity (Abramov et al., 2009), and the locus or loci of action of Aβ in HSP, therefore, remains an open question.

Other HSP-associated proteins linked to AD include cyclin-dependent kinase 5 (CDK5), a pleiotropic effector acting at various intracellular locations that has been strongly implicated in AD pathogenesis. CDK5 becomes both inappropriately activated and delocalised following Aβ exposure (Patrick et al., 1999), phosphorylating a range of targets, including tau, that contribute to the synaptic decline and eventual neuronal loss (Shukla et al., 2012). CDK5 also homeostatically regulates synaptic strength following experimental neuronal silencing, acting *via* distinct presynaptic (Kim and Ryan, 2010) and postsynaptic (Seeburg et al., 2008) mechanisms. TNFα appears to play multiple roles in AD pathogenesis since it is present at elevated levels in AD brains, and inhibiting TNFα signalling in a mouse model of AD lowered Aβ production and rescued cognitive deficits (Chang et al., 2017). TNFα of glial origin is also critical for the induction of HSP in response to chronic activity blockade (Stellwagen and Malenka, 2006). EphA4 is required for the synaptotoxic effects of oligomeric Aβ (Vargas et al., 2014), and controls excitatory synaptic strength during HSP by regulating AMPAR levels *via* CDK5 activity (Fu et al., 2007, 2011; Peng et al., 2013). Conversely, repressor element-1 silencing transcription factor (REST) appears to play a neuroprotective role in AD. REST induction in the cortex and hippocampus is a part of normal ageing, and the protein acts as a potent protector from oxidative stress and Aβ-associated toxicity. However, REST is lost in AD brains, and conditional deletion of REST in mouse brains leads to age-related neurodegeneration (Lu et al., 2014). During HSP, REST reduces the strength of excitatory synapses in response to chronic network hyperactivity by acting presynaptically to decrease the size of functional synaptic vesicle pools (Pecoraro-Bisogni et al., 2018). These examples are all mediators of classical HSP; however, an even greater variety of proteins is implicated in both AD pathogenesis and more broadly defined processes of cellular and network homeostasis, and these have been well summarised elsewhere (Jang and Chung, 2016; Styr and Slutsky, 2018).

There is, therefore, a large body of evidence to support the idea that deficits in various components of the HSP machinery are essential for AD pathogenesis, although the evidence currently falls short of definitively placing HSP in a central role. However, a recently proposed hypothesis raises exactly this possibility by invoking HSP, together with non-synaptic homeostatic mechanisms, as a functional link between the two most consistently observed early neural signatures of AD, enhanced excitatory activity in cortical and hippocampal networks and deficits in Hebbian synaptic plasticity (Styr and Slutsky, 2018).

**Figure 1:**
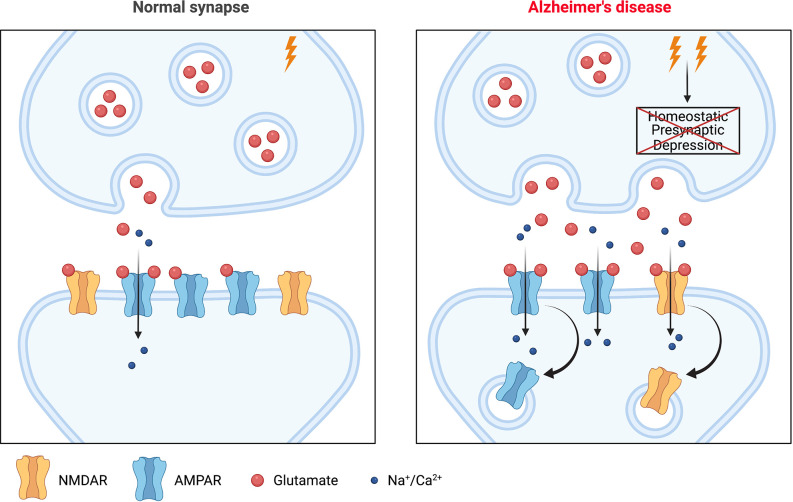
A potential role for homeostatic synaptic plasticity (HSP) in Alzheimer’s disease (AD) pathogenesis. The left panel shows a glutamatergic synapse under normal physiological conditions. The right panel shows a synapse during the early stages of AD. There is enhanced synaptic activity in affected networks, with increased synaptic glutamate release. This would normally be compensated by one or more mechanisms of HSP but if these fail, as many appear to in the context of AD, then elevated synaptic release persists and can drive a downregulation of postsynaptic strength *via* various mechanisms including postsynaptic mechanisms of HSP that remain intact, or potentially even recruitment of Hebbian plasticity (LTD). This internalization of glutamate receptors (AMPAR/NMDAR) could alter the ability of the synapse to support (further) Hebbian plasticity, with resultant effects on learning and cognition. Image created with Biorender.com. LTD, long term depression.

A region-specific increase in tonic excitatory activity is one of the earliest abnormalities that can be observed in both AD patients (Mondadori et al., 2006; Filippini et al., 2009; Sperling et al., 2009; Bateman et al., 2012; Reiman et al., 2012) and animal models of the disease (Busche et al., 2008, 2012, 2015; Rudinskiy et al., 2012; Maier et al., 2014), usually preceding the emergence of overt pathology in AD patients by years (Busche and Konnerth, 2016). It appears to play a significant role in symptomatology and possibly pathogenesis, since reducing network hyperactivity pharmacologically has been shown to improve cognitive performance in patients with mild cognitive impairment (MCI), considered to be the prodromal stage of Alzheimer’s (Bakker et al., 2012, 2015), and in AD model mice (Sanchez et al., 2012; Nygaard et al., 2015). In addition to simple hyperactivity, network hypersynchrony has been observed in AD animal models, resulting in epileptiform and seizure activity (Palop et al., 2007; Minkeviciene et al., 2009; Palop and Mucke, 2016). Consistent with this, AD patients are at elevated risk for epileptic seizures (Hauser et al., 1986; Romanelli et al., 1990), particularly those with autosomal dominant, early-onset familial AD (Palop and Mucke, 2016). The recently proposed “failure of firing homeostasis and plasticity” (FHP) hypothesis argues that such early changes are the result of a failure of homeostasis of network activity that arises when two necessary conditions have been met: firstly, some kind of perturbation of activity is imposed on susceptible brain networks, and secondly, that one or more of the core (meaning non-redundant) homeostatic mechanisms that would normally functionally compensate the perturbation are lost or compromised (Styr and Slutsky, 2018). As discussed above, the second of these criteria seems to be clearly met in AD, since several mechanisms of HSP, including a number that normally serves to downscale synaptic strength, are dysregulated. The perturbation could be any of a variety of environmental or age-related phenomena that in healthy individuals are readily compensated; in AD, drivers of the early network hyperactivity might include the build-up of Aβ, which is a positive regulator of neurotransmitter release (Abramov et al., 2009). According to the FHP hypothesis, the resultant chronically elevated network-level activity might drive maladaptive, principally postsynaptic changes *via* various mechanisms, including mechanisms of HSP that remain intact. Synapse weakening and elimination in AD might, therefore, simply represent a functional compensation that is insufficient to renormalize hyperactivity (Styr and Slutsky, 2018). These events would almost certainly impact Hebbian plasticity, both because of its mechanistic overlap with HSP, and because of the likely recruitment of metaplasticity processes, which set thresholds for Hebbian plasticity in line with the recent history of activity at the synapse (Abraham, 2008). In summary, therefore, a primary failure in mechanisms regulating HSP could lead to chronically enhanced network activity in the initial stages of the disease, which in turn could drive compensatory synapse weakening and eventual elimination, together with the plasticity deficits (impaired LTP and facilitation of LTD) that are a key substrate of cognitive decline in the early stages of AD (Mucke and Selkoe, 2012; Styr and Slutsky, 2018; Figure 1). Accordingly, the overall scheme of AD pathogenesis appears to be one in which loss of HSP function leads to deleterious consequences for affected synapses.

While this account of AD pathogenesis is highly consistent with existing experimental data obtained in a variety of model systems and with clinical observations, there is an alternative possible interpretation of the current evidence. It remains possible that postsynaptic weakening is a primary event, possibly the result of pathological signalling downstream of postsynaptic binding of pathogenic conformations of Aβ (Um et al., 2012; Kim et al., 2013), and this engages mechanisms of HSP to enhance presynaptic function and thereby synaptic activity in order to preserve synaptic strength (Taylor et al., 2020). Although this view seems much less favoured by the available evidence, it would again place HSP in an important role in pathogenesis, albeit as a maladaptive response to a pathological insult rather than as a primary, upstream event.

## Parkinson’s Disease

Like AD, Parkinson’s disease (PD) is a progressive neurodegenerative disease that shows a strong association with age. The pathology of PD is principally characterised by the degeneration of the nigrostriatal tract composed of axonal projections from dopaminergic neurons located in the midbrain substantia nigra, and is a “dying-back” axonopathy in which degeneration begins in the synaptic terminals and proceeds to the cell body (Burke and O’Malley, 2013). Clinically, PD presents with classical symptoms including bradykinesia, muscular rigidity and tremor; the first two of these are a direct result of dopaminergic neuron loss, while tremor is thought to relate to altered activity in neural circuits elsewhere in the brain (Helmich et al., 2012).

Somewhat analogous to Aβ in AD, the key trigger for PD pathogenesis is thought to be an accumulation of the protein α-synuclein (Stefanis, 2012). There is, however, a more limited body of evidence supporting the involvement of HSP in pathogenesis, at least in a cell-autonomous manner within the degenerating cell population. Physiologically, α-synuclein regulates assembly of the presynaptic SNARE protein complex that controls neurotransmitter release (Chandra et al., 2005; Burre et al., 2010), and it is thought that in the context of pathology, α-synuclein acts *via* both gain- and loss-of-function mechanisms to produce diverse effects including impairment of evoked neurotransmitter release in various PD model systems (Garcia-Reitbock et al., 2010; Nemani et al., 2010; Scott et al., 2010). The presynaptic localisation of α-synuclein and its interaction with synaptic vesicles, together with the observations that it is trafficked within neurons in an activity-dependent manner (Fortin et al., 2005), and that its expression is regulated during learning in songbirds (George et al., 1995), all argue for a physiological role in synaptic plasticity. However, this appears not to be classical HSP, but more likely the maintenance of different functional pools of synaptic vesicles that are important for healthy synaptic function in the very long term (Burre, 2015). While there is, therefore, currently no clear evidence of an effect, either loss- or gain-of-function, on HSP in PD, this remains a possibility in view of the importance of α-synuclein in synaptic regulation. Here, however, it is important to recognise that the mode of transmission at dopaminergic synapses is fundamentally different than that occurring at glutamatergic synapses of the sort affected in AD. The effects of dopamine at nigrostriatal synapses are mediated *via* volume transmission, in which the released neurotransmitter diffuses a relatively large distance to bind postsynaptic metabotropic receptors at multiple sites (Borroto-Escuela et al., 2018). This mode of synaptic transmission contrasts with the input specificity of glutamatergic synapses, which require independent regulation as well as co-regulation in functional groups of synapses across cells or networks. Indeed, it may be because of the need to balance these sometimes competing demands that HSP plays such an important role at glutamatergic synapses (Vitureira and Goda, 2013) and, conversely, may be less significant at dopaminergic synapses.

**Figure 2:**
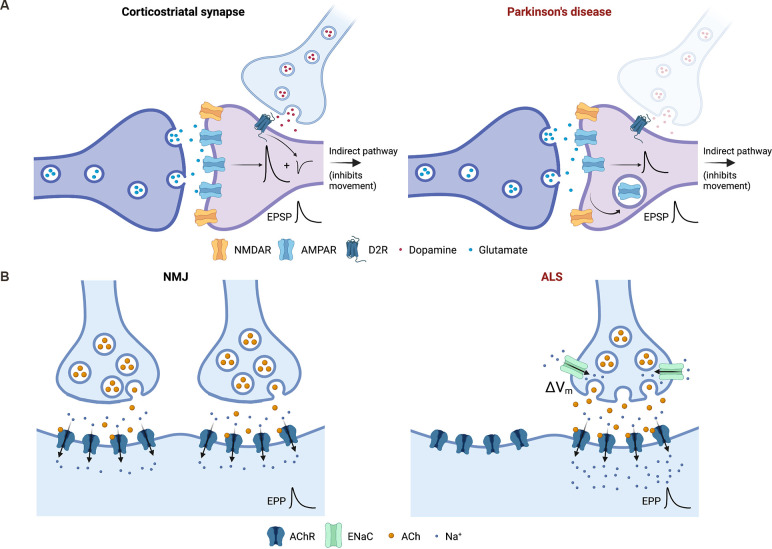
Induction of homeostatic synaptic plasticity (HSP) can support the functioning of neuronal networks and/or populations of synapses in the context of synaptic degeneration. **(A)** Heterosynaptic and cell non-autonomous induction of HSP supports the function of basal ganglia circuits following degeneration of dopaminergic synapses in Parkinson’s disease (PD). One example of this is shown involving synapses of the indirect pathway, which serves to negatively regulate voluntary movement. In models of PD, the strength of glutamatergic corticostriatal synapses onto medium spiny neurons is scaled down *via* mechanisms of HSP to compensate for the loss of D2 receptor-dependent inhibition (conceptually represented in the left panel as an inhibitory potential) of the same neuron by the degenerating nigrostriatal dopaminergic projections (right panel). Thus, heterosynaptic induction of HSP ensures that the overall strength of excitatory postsynaptic potentials (EPSPs) measured at the medium spiny neuron soma is preserved. **(B)** Induction of HSP at the degenerating neuromuscular junction (NMJ) supports motor function and opposes disease progression in amyotrophic lateral sclerosis (ALS) pathogenesis. The left panel shows the NMJ under physiological conditions. The right panel represents early stage ALS, in which innervation of the muscle and motor function is compromised by the loss of motor neuron synapses. This results in the induction of presynaptic HSP in remaining synaptic terminals *via* the membrane insertion of epithelial sodium channels (ENaC) that constitutively depolarise the presynaptic membrane (ΔV_m_) and augment neurotransmitter release. This serves to: (1) preserve the strength of degenerating synapses; and/or (2) maintain normal or near-normal levels of postsynaptic depolarisation and end plate potentials (EPP) despite the loss of synaptic terminals, as shown here. Image created with Biorender.com.

While there is little evidence for a role for HSP at the degenerating nigrostriatal synapses, there is evidence that dopamine depletion triggers the induction of a variety of heterosynaptic and cell non-autonomous homeostatic mechanisms within the striatum in order to preserve the function of basal ganglia circuits of which the degenerating synapses are part. These adaptations are principally seen in various classes of spiny projection neurons and include structural changes, such as changes in the density of dendritic spines (Stephens et al., 2005), changes in cellular excitability (Fieblinger et al., 2014) and changes in the ability of corticostriatal synapses to support Hebbian plasticity (Thiele et al., 2014). There is also evidence for changes in basal strength of striatal synapses following dopamine depletion, which are likely to represent typical HSP (Villalba and Smith, 2018). In particular, there is a specific reduction in the strength of glutamatergic corticostriatal synapses on to D2 receptor-expressing spiny neurons of the indirect pathway (Suarez et al., 2016), the inhibitory arm of the system that represents the major regulator of the initiation and amplitude of voluntary movements. This change appears to require the local upregulation of TNFα, an established mediator of HSP (Lewitus et al., 2014), and serves to maintain the strength of output from spiny neurons that would otherwise be pathologically enhanced by the loss of heterosynaptic D2R-mediated inhibition accompanying the loss of dopaminergic afferents (Figure 2A). In addition, there is a loss of both synaptic strength and connectivity amongst the recurrent collateral synapses that mediate inhibitory synaptic activity amongst the spiny neurons themselves (Taverna et al., 2008), although the functional significance of this is less clear.

These observations, therefore, suggest another potential role for HSP in neurodegenerative disease, quite distinct from its involvement in driving degeneration through loss of function as in AD. In the context of PD, HSP can be recruited in a heterosynaptic or cell non-autonomous manner at various loci of control within basal ganglia circuits to resist the effects of synaptic/neuronal loss, specifically the loss of dopaminergic nigrostriatal projections, elsewhere.

## Huntington’s Disease

Huntington’s disease (HD) is an inherited autosomal dominant neurodegenerative disorder caused by an elongation of the CAG repeat region of the gene encoding the huntingtin (Htt) protein (MacDonald et al., 1993). The disease usually manifests in middle age and is characterised initially by loss of motor coordination, frequently accompanied by evidence of cognitive impairment and psychiatric changes. These early symptoms develop into a hyperkinetic movement disorder and, usually, an overt dementia. The rate of decline and severity of symptoms is correlated with the length of the pathogenic CAG repeat. Like PD, HD is a condition that affects the basal ganglia, and the motor symptoms are a direct result of degeneration of a specific class of striatal spiny projection neurons, an effect that is thought to be mediated *via* a toxic gain-of-function of the mutant Htt (Ross and Tabrizi, 2011).

In common with other neurodegenerative diseases HD does, therefore, exhibit a degree of selective neuronal vulnerability (Fu et al., 2018). However, a variety of neuronal populations may be involved, which is perhaps expected since HD is a genetic disease, and all cells will express the toxic mutant Htt protein. Although the degeneration of striatal spiny neurons is perhaps the best-recognized feature of HD pathology, cortical neurons are also affected relatively early in the course of the disease, accounting for the high incidence of cognitive and psychiatric symptoms in HD (Ross and Tabrizi, 2011). Cortical neurons are much more accessible and tractable for study than striatal neurons and have yielded the strongest evidence to suggest dysregulation of HSP in HD. In one study, cultured cortical neurons from HD model mice were shown to lack the ability to induce HSP in response to network silencing, a phenotype which could be rescued by enhancing BDNF signalling (Smith-Dijak et al., 2019). *In vivo*, loss of HSP is a likely explanation for the failure of dendritic spines in the barrel cortex to adapt to the loss of sensory input as expected following whisker trimming (Murmu et al., 2015). There is also some evidence from human HD patients, with data from transcranial magnetic stimulation of the motor cortex revealing various abnormalities including impairments in LTP and LTD-like plasticity at cortical synapses that might partly reflect defective homeostatic scaling processes (Calabresi et al., 2016).

In addition, there is some indirect evidence for a role for HSP in Huntington’s disease (HD), although this is less compelling. A study of the Htt interactome and of perturbation genes relevant for HD pathology endpoints showed enrichment of both datasets for HSP-related genes, suggesting that interactions of mutant Htt with HSP-related proteins may contribute to pathogenesis or progression in HD (Wang et al., 2017). Furthermore, the microRNA miR-485, expression of which is dysregulated in HD, plays a key regulatory role in HSP, again raising the possibility of some overlap between these processes (Cohen et al., 2011). Overall, therefore, the picture emerging is one of loss and/or dysregulation of function in pathways mediating HSP, although the pathogenic significance of this is not yet clear, as these effects remain relatively little explored. Given the importance of HSP in maintaining healthy neuronal and network function, it seems likely that these deficits will play a role in pathogenesis or progression of HD, as well as possibly in the generation of motor symptoms, and HD would in this case belong to the broad functional class of HSP involvement as AD, where changes in HSP are deleterious. It does, however, remain possible, if unlikely, that the dysregulation of HSP is merely a bystander phenomenon that contributes little to pathogenesis, and further work will be required to resolve these issues.

## Amyotrophic Lateral Sclerosis

Amyotrophic lateral sclerosis (ALS) is a progressive neurodegenerative disease principally affecting upper and lower motor neurons and presenting as progressive motor deficits that develop over weeks or months. In later stages, there may also be cortical involvement with cognitive symptoms (van Es et al., 2017). Like other neurodegenerative diseases, the aetiology is not well understood but appears to be a complex mix of genetics and environmental factors. The greatest insights into pathogenesis thus far have come from the identification of a number of genes associated with familial variants of ALS, which group functionally into three main categories: RNA biology, protein turnover and axonal transport, suggesting that deficits in these processes play a causal role in pathogenesis (Renton et al., 2014). The discovery of causative genes has also assisted greatly with the production of suitable animal models of ALS for further mechanistic studies.

Because the most prominent feature is usually lower motor neuron degeneration with resultant muscular weakness, ALS is frequently thought of as a disease of the neuromuscular junction (NMJ; van Es et al., 2017). There is evidence in support of potential adaptive or homeostatic changes at NMJs in various animal models of ALS, and some in particular demonstrate enhanced spontaneous neurotransmission (Dzieciolowska et al., 2017; Bose et al., 2019), although it is not clear whether this is functionally significant or beneficial.

One intriguing feature of ALS, which is by no means unique to this disease, is that a significant loss of synapses can occur in affected regions, principally the NMJ, prior to the onset of symptoms (Moloney et al., 2014). This observation suggests that endogenous mechanisms may, to some degree, be recruited to preserve function in the face of reduced synaptic number, although until recently the existence and identity of such mechanisms had never been confirmed. In 2020, a study initiated at the *Drosophila* NMJ showed that classical presynaptic HSP is induced at degenerating NMJs and that it functionally opposes the effects of motor neuron degeneration and loss (Orr et al., 2020). Presynaptic HSP is known to depend on the presynaptically localised epithelial Na^+^ channel, ENaC (Younger et al., 2013), which was the basis for most of the manipulations used in this study. The authors went on to demonstrate that presynaptic HSP is induced similarly in a mouse model of motor neuron degeneration (Orr et al., 2020), supporting its relevance in mammalian systems. These exciting results establish a new paradigm for HSP in neurodegenerative disease whereby it is induced within the degenerating population of synapses themselves to functionally oppose the effects of disease progression (Figure 2B). It remains unclear whether this process is initiated within individual terminals during the process of degeneration in order to augment their own declining function, or whether it may be initiated by orphan postsynaptic elements in regions of synapse loss to enhance release from remaining terminals nearby. Alternatively, both of these mechanisms may operate, and further work will be required to resolve the various possibilities. In addition to the functional rescue, Orr et al. demonstrated that the induction of HSP at NMJs actually arrests degeneration. They hypothesize that this might be because the loss of synaptic transmission could lead to a loss of trophic support (growth and survival factors) normally released by the innervated muscle (Orr et al., 2020).

## Conclusions

The body of evidence in support of a role for HSP in a variety of neurodegenerative diseases is growing steadily. What, then, is the nature of this role? We have presented here an overview of the currently available evidence, which suggests that the significance of HSP may be distinctly different in each of the major diseases associated with neurodegeneration.

In this context, HSP appears able to function in three broad ways, notwithstanding that there may be more yet to be recognised. The first is a direct involvement in pathogenesis, usually when the failure of one or more mechanisms of HSP in degenerating synapses leads to consequences that drive the development of disease. A substantial body of evidence suggests that this is a critical mechanism underlying Alzheimer’s disease, and it also seems likely to be relevant to Huntington’s disease, although here there is currently much less evidence to support the conclusion. Secondly, HSP may be induced in a heterosynaptic or cell non-autonomous manner in connected neurons to preserve the function of networks of which the degenerating synapses are part, as may happen in Parkinson’s disease. Thirdly and finally, HSP may be recruited within degenerating populations of synapses themselves to oppose functional decline and even mitigate synapse loss, as in amyotrophic lateral sclerosis.

Recognition of the diverse manifestations of HSP in the disease context will aid the development of new therapeutic approaches, which are acutely needed. These may aim to restore or replace a dysfunctional protein or pathway, or to further augment a beneficial homeostatic response. It may also be possible to initiate homeostatic responses in cells in which these may be dormant or not otherwise induced. Such an approach has recently been validated in a *Drosophila* model of *C9orf72*-associated motor neuron loss in which presynaptic HSP is normally inactive at degenerating NMJs, but can be experimentally induced to restore synaptic strength (Perry et al., 2017).

## Author Contributions

HT and AJ conceived the review and wrote and reviewed the manuscript. All authors contributed to the article and approved the submitted version.

## Conflict of Interest

The authors declare that the research was conducted in the absence of any commercial or financial relationships that could be construed as a potential conflict of interest.

## Publisher’s Note

All claims expressed in this article are solely those of the authors and do not necessarily represent those of their affiliated organizations, or those of the publisher, the editors and the reviewers. Any product that may be evaluated in this article, or claim that may be made by its manufacturer, is not guaranteed or endorsed by the publisher.
